# Circulating miR-223-3p as an Independent Biomarker of Recurrent Thrombotic Risk After Ischemic Stroke

**DOI:** 10.3390/biomedicines13122961

**Published:** 2025-12-01

**Authors:** Bence Balczó, Katalin Maricza, Krisztina Molnár, Zsuzsanna Elek, Zsófia Bánlaki, Réka Kovács-Nagy, Gergely Keszler, Zsolt Rónai, Abigél Molnár, Tihamér Molnár

**Affiliations:** 1 Department of Pediatric Care, Csolnoky Ferenc Hospital, 8200 Veszprém, Hungary; balczoben@gmail.com; 2 Department of Molecular Biology, Institute of Biochemistry and Molecular Biology, Semmelweis University, 1094 Budapest, Hungary; maricza.katalin@stud.semmelweis.hu (K.M.); molnar.krisztina@stud.semmelweis.hu (K.M.); elek.zsuzsanna@semmelweis.hu (Z.E.); banlaki.zsofia@semmelweis.hu (Z.B.); kovacs-nagy.reka@semmelweis.hu (R.K.-N.); keszler.gergely@semmelweis.hu (G.K.); ronai.zsolt@semmelweis.hu (Z.R.); 3 Central Electron Microscope Laboratory, Medical School, University of Pécs, 7624 Pécs, Hungary; molnar.abigel@pte.hu; 4 Department of Anesthesiology and Intensive Care, Medical School, University of Pécs, 7624 Pécs, Hungary

**Keywords:** stroke, thrombotic events, platelet, miRNAs, risk analysis

## Abstract

**Background:** Circulating microRNAs (miRNAs) have emerged as potential biomarkers of platelet reactivity and thrombotic risk. Among them, miR-223-3p regulates P2Y12 receptor expression and may influence response to antiplatelet therapy. This study aimed to evaluate the prognostic value of selected circulating miRNAs in post-stroke patients receiving antiplatelet treatment. **Methods:** Sixty ischemic stroke survivors were prospectively enrolled and followed for 18 months for recurrent vascular events (stroke, transient ischemic attack, or myocardial infarction). Plasma levels of miR-126-3p, miR-223-3p, miR-24-3p, and miR-199a-5p were quantified using reverse transcription real-time PCR. Clinical data, antiplatelet regimen, statin use, and Essen Stroke Risk Scores (ESRS) were recorded. Logistic regression was applied to identify independent predictors of thrombotic events. **Results:** Expression of all examined miRNAs differed significantly across treatment groups. The dual antiplatelet therapy (DAPT) group showed the highest levels of miR-126-3p and miR-199a-5p (*p* < 0.01). Within the statin-naïve DAPT subgroup, lower miR-199a-5p levels (*p* < 0.001) were observed among patients who experienced ischemic events (n = 7/60; 12%; stroke = 4, TIA = 2, ACS = 1) during 18 months of follow-up. In multivariate analysis, reduced miR-223-3p remained the only independent predictor of recurrent thrombotic events (OR 1.18, 95% CI 1.01–1.37, *p* = 0.036), independent of ESRS and platelet reactivity. Elevated miR-126-3p and miR-199a-5p were associated with favorable treatment response, particularly among statin users. **Conclusions:** This study identifies low circulating miR-223-3p as an independent biomarker of thrombotic risk in post-stroke patients, potentially reflecting enhanced platelet activation via P2Y12 signaling. In contrast, higher miR-126-3p and miR-199a-5p levels may indicate more effective antiplatelet response. These findings support the potential utility of miRNA profiling for individualized antiplatelet therapy and long-term risk stratification after ischemic stroke.

## 1. Introduction

### 1.1. Antiplatelet Therapy and Stroke Prevention

Stroke remains a leading cause of mortality and long-term disability worldwide, accounting for over 10% of all deaths [1]. Despite advances in prevention and acute care, the global burden of stroke remains high, with approximately 40,000 new cases annually in Hungary alone, of which nearly 10,000 are fatal [2,3].

Antiplatelet therapy is a cornerstone of secondary prevention in ischemic stroke, aiming to reduce recurrent thrombotic events. Commonly prescribed agents include aspirin, a cyclooxygenase (COX) inhibitor; clopidogrel, a P2Y12 receptor antagonist; and dual antiplatelet therapy (DAPT) combining both mechanisms [4]. However, a substantial proportion of patients—estimated between 4% and 30%—exhibit low or no responsiveness to antiplatelet drugs, leading to increased risk of recurrence and poorer outcomes [5,6,7].

### 1.2. Mechanisms Underlying Antiplatelet Non-Responsiveness

Variability in antiplatelet response has been attributed to genetic, metabolic, and platelet-intrinsic factors [8]. Platelets are highly heterogeneous in age, size, and reactivity. An elevated immature platelet fraction (IPF) has been associated with high platelet reactivity (HPR) and reduced clopidogrel sensitivity [9]. Platelet populations are heterogeneous in terms of age, size, reactivity, and response to antiplatelet agents [10,11]. An elevated immature platelet fraction (IPF) has been associated with reduced responsiveness to clopidogrel [12]. Molnár et al. [13] examined platelet behavior in post-ischemic stroke patients using a modified one-hour gravity sedimentation test, analogous to the Erythrocyte Sedimentation Rate (ESR). Their study revealed that immature, more reactive platelets tend to migrate upward, in contrast to the downward sedimentation of erythrocytes and mature platelets. This observation led to the development of a novel parameter—the Platelet Antisedimentation Rate (PAR)—which reflects increased platelet reactivity and may serve as a predictive marker for reduced clopidogrel responsiveness. The contribution of age-related platelet-derived microRNAs (miRNAs) to antiplatelet therapy non-responsiveness represents a clinically significant yet underexplored area of research. Growing scientific interest has also focused on the broader roles of platelets beyond hemostasis—particularly their involvement in inflammation resolution, vascular protection, and tissue repair [14].

### 1.3. Platelet-Derived MicroRNAs and Their Functional Roles

Although anucleate, platelets inherit a repertoire of functional microRNAs (miRNAs) from megakaryocytes [11]. These small non-coding RNAs regulate gene expression post-transcriptionally by binding to target mRNAs, thereby influencing platelet activation, aggregation, and intercellular communication. Among the most studied platelet-derived miRNAs, miR-223-3p and miR-126-3p play central roles in platelet function and vascular homeostasis. MiR-223-3p targets the P2Y12 receptor, potentially affecting clopidogrel binding efficacy [15], while miR-126-3p modulates platelet–collagen interactions through ADAM9 regulation [16]. Other miRNAs, such as miR-199a-5p and miR-24-3p, influence inflammatory and apoptotic pathways and may contribute to neuronal recovery and vascular repair following ischemic injury [17,18,19]. The mature miRNAs are subsequently packaged into platelets and retained in the circulating blood [20].

MiRNAs regulate gene expression post-transcriptionally by binding to complementary sequences on target mRNAs, thereby inhibiting their translation [21]. Some of these platelet-derived miRNAs are also secreted into the bloodstream, becoming part of the circulating miRNA pool [22]. Among the most studied in cardiovascular diseases are miR-126-3p and miR-223-3p [23]. MiR-223-3p has been shown to downregulate the P2Y12 receptor, potentially reducing clopidogrel binding efficacy [24]. MiR-126-3p is a potent regulator of platelet activity; it not only influences P2Y12 receptor expression but also downregulates ADAM9 in megakaryocytes—a gene involved in platelet–collagen interactions [25,26].

### 1.4. Current Evidence and Knowledge Gaps

Despite growing evidence linking circulating miRNAs to cardiovascular and cerebrovascular disorders, findings across studies are inconsistent. For example, conflicting results have been reported for miR-223-3p levels in clopidogrel non-responders, possibly due to methodological differences in sample handling and normalization [27,28,29]. Moreover, most studies have focused on coronary artery disease, while data in post-stroke populations remain scarce.

Critically, the relationship between platelet-derived miRNAs, platelet heterogeneity markers (e.g., PAR, IPF), and antiplatelet non-responsiveness has not been systematically investigated in ischemic stroke. Understanding these associations could help identify patients at higher thrombotic risk and guide more personalized antiplatelet strategies.

Moreover, future research should prioritize the use of miRNA panels over single markers to enhance the predictive accuracy for high on-treatment platelet reactivity [19].

In addition to miR-126 and miR-223-3p, other miRNAs such as miR-199a-5p and miR-24-3p have emerged as key regulators of inflammation, apoptosis, and oxidative stress. MiR-199a-5p influences hypoxia-inducible factor 1α (HIF-1α) and mTOR signaling pathways, with its downregulation associated with improved neuronal recovery following stroke [30]. MiR-24-3p modulates genes related to apoptosis, inflammation, and vascular permeability, and has been linked to neuroprotection in cerebral ischemia [31]. Its dynamic expression across different stroke phases suggests a potential role in post-stroke tissue remodeling [32].

### 1.5. Aim of This Study

This study aimed to explore the expression of selected circulating miRNAs—miR-223-3p, miR-126-3p, miR-199a-5p, and miR-24-3p—in post-stroke patients receiving antiplatelet therapy. By correlating miRNA profiles with platelet reactivity parameters, we sought to identify potential biomarkers predictive of thrombotic risk and treatment response. We hypothesize that distinct miRNA expression patterns are associated with high platelet reactivity and could serve as novel tools for individualized antiplatelet therapy.

## 2. Materials and Methods

### 2.1. Study Design and Location

The study protocol was approved by the Regional and Institutional Research Ethics Committee of the University of Pécs Clinical Centre (Ref. number: 6735; Clinical Trial ID: NTC03679858). Written informed consent was obtained from all participants.

A total of 60 patients [mean age: 68 ± 11 years; male: 36] receiving antiplatelet therapy for secondary stroke prevention were prospectively enrolled. Patients were divided into three equal subgroups based on their treatment regimen: (i) AM group (n = 20): 100 mg aspirin once daily; (ii) CM group (*n* = 20): 75 mg clopidogrel once daily; (iii) DAPT group (*n* = 20): combination of aspirin and clopidogrel. Patients were regularly followed up at the outpatient neurology clinic. All were instructed to take their prescribed medication at least 2 h prior to blood sampling. Exclusion criteria were as followings: (i) Irregular medication adherence; (ii) Congenital platelet disorders; (iii) Congenital coagulation disorders (e.g., hemophilia); (iv) Anemia; and (v) Use of anticoagulant therapy (e.g., warfarin, NOACs).

Comorbidities, medications, and smoking status were documented. Clinical risk factors, secondary prevention strategies, Essen Stroke Risk Score (ESRS), and clinical outcomes during an 18-month follow-up period were also recorded. All follow-up data were obtained from an official electronic healthcare database, which includes outpatient records and hospital discharge summaries. The primary endpoint was the occurrence of thrombotic events (e.g., stroke, TIA, acute coronary syndrome, ACS).

### 2.2. Subjects (Inclusion and Exclusion Criteria) and Blood Sampling

Venopuncture after fasting was performed from the cubital vein after short time strangulation of the arm with 21 G BD vacutainer needle. The total blood count and specific platelet parameters were measured of 1 × 3 mL blood samples taken into one vacutrainer with EDTA (REF: 368856, 5.4 md EDTA). Also, blood for platelet aggregometry was taken into 2 × 3 mL hirudin containing tube for Multiplate^®^ Analyzer (Roche Diagnostics, Mannheim, Germany).

### 2.3. Measurements of Platelet Function [Electrical Impedance Aggregometry]

Platelet function test from the whole blood was performed from a hirudin containing tube with a Multiplate^®^ Analyzer (Roche Diagnostics, Mannheim, Germany). Platelet aggregometry was universally performed 20 min after blood sampling. Stimulation protocols: CM group: 6.5 μM adenosine diphosphate (ADP); AM group: 6.5 μM arachidonic acid (AA); DAPT group: both ASPItest (AA) and ADPtest were conducted separately. Aggregation was expressed as the Area Under the Curve (AUC) in aggregation units × time (minutes). According to the manufacturer: in case of ADP stimulation, the normal AUC range = 53–122, while in case of AA stimulation, the AUC range = 75–136 [33,34]. Patients were classified as responder (AUC < 53) and non-responder subjects (NR) (AUC ≥ 53) based on the ADP test in the CM group [13].

### 2.4. Essen Stroke Risk Score

The Essen Stroke Risk Score (ESRS) is a validated clinical prediction tool developed to estimate the long-term risk of recurrent ischemic stroke and major vascular events in patients with a prior ischemic stroke or transient ischemic attack (TIA) [35]. It was initially derived and validated in large population-based cohorts, including data from the CAPRIE and ESPRIT trials, and has since been widely utilized in both clinical and research settings for vascular risk stratification. It analyzes seven clinical variables (age ≥ 65 years, arterial hypertension, diabetes mellitus, previous myocardial infarction, other cardiovascular diseases (including heart failure and peripheral arterial disease), current or past smoking, previous stroke or TIA) each one count as one point to the overall score. The total score ranges from 0 to 9, with higher scores reflecting increased vascular comorbidity and a greater predicted risk of stroke recurrence. Patients with an ESRS ≥ 3 have been shown to have significantly higher annual rates of recurrent stroke and vascular events compared to those with lower scores [35].

### 2.5. Micro-RNA Methodology

**Figure 1:**
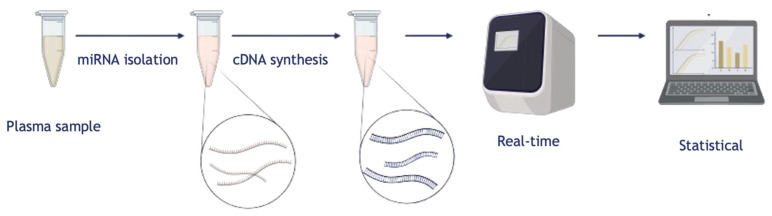
The process of RNA isolation and Real-time quantitative PCR. After total RNA-isolation cDNA was transcribed for subsequent qPCR-analysis using Sybr Green fluorescent dye. Expression levels of the miRNAs were determined by the ΔC_T_-method.

After the one-hour gravity sedimentation method 3 × 1 mL plasma were separated and centrifugated from each patient regarding the whole, the upper and lower plasma fraction, respectively. Phase separation was performed by centrifugation for 15 min at 12,000× *g*, 4 °C, followed by several centrifugation steps at 8000× *g* for 1 min at room temperature according to the manufacturer’s protocol. Subsequently the samples were stored under −70 °C degree for further miRNA studies. The total RNA was isolated from the whole, the upper and lower plasma fraction at the Semmelweis University in the Department of Molecular Biology using Qiagen Mini Kit, (QIAGEN, Hilden, Germany) cDNA was transcribed from total miRNA by miRNA 1st-Strand cDNA Synthesis Kit. Expression levels of target miRNAs were quantitated by Sybr Green based ΔCT real-time PCR method. For endogenous control miR-484 and miR-23a-3p were selected based on literature data [36]. In case of each sample, multiple technical parallel measurements were conducted (Figure 1).

### 2.6. Statistics

Statistical analysis was conducted using SPSS 19.0 (SPSS Inc., Chicago, IL, USA). Categorical variables were expressed as absolute and relative frequencies (counts and percentages). Comparisons of miRNA expression between groups were performed using the Mann–Whitney U test as well as One-way ANOVA test. A *p*-value < 0.05 was considered statistically significant. Binary logistic regression was used to assess independent predictors of thrombotic events, adjusting for covariates such as age, sex, ESRS, and residual platelet reactivity (based on Multiplate).

## 3. Results

### 3.1. Study Population

A total of 60 convalescent ischemic stroke patients were enrolled and evenly distributed across three treatment groups: aspirin monotherapy (AM, *n* = 20), clopidogrel monotherapy (CM, *n* = 20), and dual antiplatelet therapy (DAPT, *n* = 20).

Baseline demographic and clinical characteristics are summarized in Table 1. The mean age of participants was 68 ± 11 years, with 60% being male. No significant intergroup differences were observed regarding age, sex, smoking status, or vascular risk factors. Hypertension (92%) and diabetes mellitus (42%) were the most frequent comorbidities. The mean Essen Stroke Risk Score (ESRS) was 3.1 ± 1.2 across groups, indicating a moderate to high vascular risk profile.

### 3.2. Platelet Function Testing

Multiplate^®^ aggregometry confirmed expected pharmacologic responses across treatment regimens. In the ASA group, arachidonic acid (AA)–induced aggregation was effectively suppressed, with AUC values below the manufacturer’s threshold for non-responsiveness. In the clopidogrel group, adenosine diphosphate (ADP)–induced aggregation showed greater variability; 25% (5/20) of patients met the criteria for high on-treatment platelet reactivity (AUC ≥ 53). The DAPT group exhibited dual inhibition, with significantly lower AA- and ADP-induced aggregation compared to both monotherapy groups (*p* < 0.01).

### 3.3. Antiplatelet Regimen and Circulating miRNA Levels

**Figure 2:**
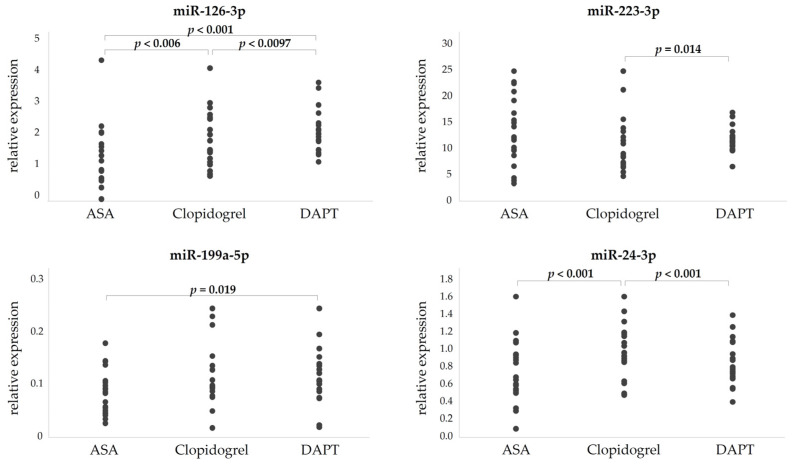
Expression levels of microRNAs in different antiplatelet subgroups.

**Figure 3:**
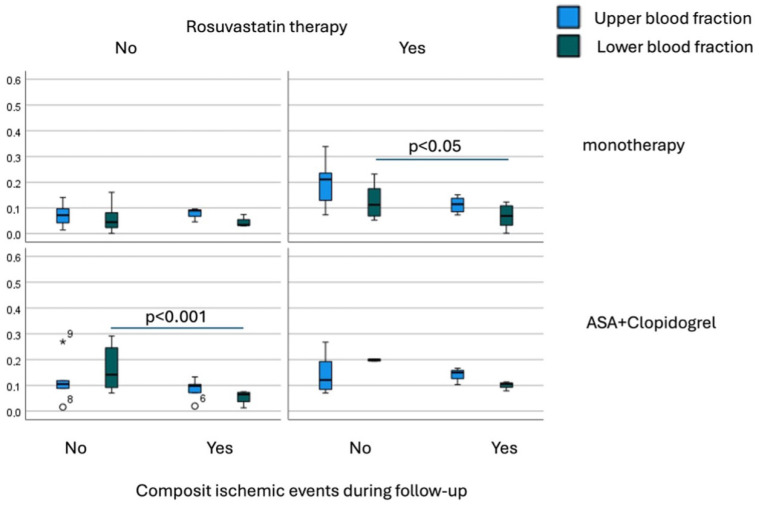
Expression of miR-199a-5p in subgroups dichotomized by either statin or antiplatelet therapy in patients with and without ischemic events during follow-up. Asterix and circles indicate outliers.

Distinct expression profiles of the studied miRNAs were observed among treatment groups (Figure 2). Compared to monotherapy, DAPT was associated with significantly higher plasma levels of miR-126-3p (*p* < 0.001) and miR-199a-5p (*p* = 0.002). miR-223-3p was moderately elevated in DAPT versus clopidogrel monotherapy (*p* = 0.014). miR-24-3p levels were significantly higher in both ASA and DAPT groups compared to clopidogrel (*p* < 0.001). Within the statin-naïve DAPT subgroup, lower miR-199a-5p levels (*p* < 0.001) were observed among patients who experienced ischemic events during follow-up, suggesting potential prognostic value (Figure 3).

### 3.4. Independent Predictors of Ischemic Events

Binary logistic regression including age, sex, ESRS, platelet reactivity, and miRNA expression identified miR-223-3p (cut-off ≤ 10.0) as an independent predictor of thrombotic events (TIA, stroke, ACS) during the 18-month follow-up (OR 1.18, 95% CI 1.01–1.37, *p* = 0.036), independent of the antiplatelet regimen.

### 3.5. Interaction with Statin Therapy

Next expression levels of target miRNAs were quantified separately from whole plasma, as well as from the upper and lower plasma fractions. In a subgroup of patients receiving both statin therapy and antiplatelet monotherapy, miR-199a-5p expression in the lower plasma fraction was significantly reduced in those who experienced ischemic events (*n* = 7) compared to patients without complications (*n* = 53) (*p* < 0.05). Conversely, in patients on dual antiplatelet therapy (DAPT) who were not taking statins, miR-199a-5p was significantly overexpressed in the lower fraction among those who remained free of ischemic events during the follow-up period (Figure 3) (*p* < 0.001).

### 3.6. Clinical Outcomes

During 18 months of follow-up, seven thrombotic events occurred (stroke = 4, TIA = 2, myocardial infarction, ACS = 1). Five of these events (71%) occurred in patients classified as non-responders by aggregometry. Logistic regression confirmed that both high residual platelet reactivity (OR 3.6; 95% CI 1.2–10.8; *p* = 0.02) and higher ESRS (OR 1.8; 95% CI 1.1–3.0; *p* = 0.04) independently predicted recurrent ischemic events.

## 4. Discussion

This study provides novel insights into the modulation of antiplatelet therapy efficacy by circulating microRNAs (miRNAs) in post-stroke patients, underscoring their potential role as emerging biomarkers for ischemic risk stratification and therapeutic response. Our results demonstrate that plasma levels of miR-126-3p, miR-199a-5p, and miR-223-3p differ significantly according to the type of antiplatelet therapy and associate with clinical outcomes during an 18-month follow-up period.

The elevated expression of miR-126-3p and miR-199a-5p observed in patients on dual antiplatelet therapy (DAPT) is consistent with prior research highlighting their crucial roles in vascular homeostasis and inflammation regulation. MiR-126-3p is a well-established modulator of endothelial function and angiogenesis, promoting vascular repair and inhibiting platelet activation through targeting key signaling pathways [37,38]. Our finding that miR-126-3p levels were significantly lower in DAPT patients who experienced ischemic events reinforces its protective role and supports its potential utility as a biomarker for effective platelet inhibition. This aligns with previous studies demonstrating miR-126-3p’s involvement in the modulation of platelet reactivity [39,40].

Similarly, miR-199a-5p, known to regulate hypoxia-inducible factor 1α [HIF-1α] and mTOR signaling, has been implicated in controlling inflammatory responses, apoptosis, and oxidative stress during ischemic injury [41,42]. The observed reduction in miR-199a-5p expression in statin-treated patients who developed ischemic events, contrasted with its elevation in non-statin-treated patients without complications, suggests a complex interaction between statin therapy, inflammation modulation, and miRNA regulation. This may reflect the pleiotropic effects of statins on endothelial function and inflammatory pathways, which are critical determinants of stroke recovery [43,44]. However, our clinical data alone cannot determine whether statins directly modulate miRNA expression, whether antiplatelet therapy modifies this effect, or whether both treatments influence upstream biological processes that secondarily alter circulating miRNA profiles. Definitive clarification of these mechanisms would require targeted experimental studies.

Importantly, miR-223-3p was identified as an independent predictor of thrombotic events, with decreased plasma levels significantly associated with recurrent ischemic complications, regardless of antiplatelet regimen. MiR-223-3p plays a crucial role in regulating platelet function through downregulation of the P2Y12 receptor, a primary target of clopidogrel [39,45]. Reduced circulating miR-223-3p has been linked to high platelet reactivity and poorer clinical outcomes in acute coronary syndrome patients [46,47], supporting our findings that repression of miR-223-3p may compromise antiplatelet efficacy and increase thrombotic risk [48].

Notably, previous studies have reported inconsistent results for miR-223-3p expression. For example, elevated miR-223-3p levels were observed in patients with ischemic events shortly after the acute phase, whereas others found decreased expression in the chronic phase associated with higher platelet reactivity and worse outcomes [47]. These discrepancies may stem from several factors, including differences in patient populations (acute versus chronic stroke phase, comorbid conditions such as diabetes or coronary artery disease), sample types (serum, platelet-rich or platelet-poor plasma), and assay methodologies (RNA extraction and normalization protocols). In addition, timing of sampling relative to antiplatelet initiation and drug exposure heterogeneity may markedly influence circulating miR-223-3p levels, given its rapid turnover in platelets. Our study focused on stable post-stroke patients under long-term secondary prevention, which may better reflect steady-state miRNA modulation by chronic therapy rather than acute-phase responses.

Taken together, this microRNA directly targets P2Y12 receptor mRNA, and its downregulation leads to enhanced P2Y12 expression and increased platelet reactivity. Additional targets such as FBXW7 and IGF1R link miR-223-3p to granule release and vascular inflammation, suggesting a broader regulatory role in thrombus formation. Our results therefore support the concept that miR-223-3p acts as a molecular modulator of platelet responsiveness, providing a mechanistic basis for its association with recurrent ischemic events.

While miR-24-3p showed differences between therapy groups, its role remains less clearly defined. MiR-24-3p has been implicated in regulating apoptosis, inflammation, and vascular permeability, and may contribute to neurovascular remodeling post-stroke [32]. Although further studies are required to clarify its clinical utility, miR-24-3p’s modulation in our cohort suggests it could be part of the complex miRNA network influencing vascular and platelet biology.

Our findings highlight the potential of miRNA profiling to complement established clinical risk assessment tools such as the Essen Stroke Risk Score (ESRS). Notably, additional insights were obtained by measuring miRNAs not only from whole plasma but also from the upper and lower fractions separated via the one-hour gravity sedimentation method. Unlike static clinical scores, miRNAs capture dynamic molecular processes related to platelet function, endothelial integrity, and inflammation. This provides a more nuanced and responsive approach to individualized risk stratification and therapeutic optimization [49,50].

Looking ahead, expanding the molecular scope of future studies may further enhance understanding of platelet regulation and ischemic risk. In particular, additional microRNAs—including miR-21, miR-30b, and miR-22—have been identified as important modulators of platelet activation, endothelial signaling, and vascular inflammation. MiR-21 regulates pathways linked to platelet reactivity and vascular remodeling, while miR-30b contributes to endothelial cell responses and apoptotic balance, and miR-22 influences cardiomyocyte survival and post-ischemic vascular integrity. These miRNAs operate within broader regulatory networks affecting thrombus formation and cerebrovascular outcomes, as highlighted in recent mechanistic studies [51,52]. Incorporating such additional regulatory pathways in future research may help delineate the multi-layered miRNA interactions underlying antiplatelet therapy responsiveness and could ultimately support the development of more comprehensive, personalized strategies for secondary stroke prevention.

Limitations of this study include its relatively small sample size and single-center design, which may affect the generalizability of these preliminary results. Therefore, it requires validation in a larger, independent cohort. Additionally, variability in miRNA measurement techniques and pre-analytical factors remain challenges for clinical translation. Despite these constraints, the prospective design and long-term follow-up strengthen the reliability of the observed associations.

In summary, our data may suggest that increased expression of miR-126-3p and miR-199a-5p may enhance antiplatelet responses and improve outcomes in post-stroke patients, particularly in the context of statin use. Conversely, decreased miR-223-3p independently predicts recurrent thrombotic events, highlighting its protective role in long-term therapy. These miRNAs warrant further validation in larger cohorts and mechanistic studies, with the ultimate goal of integrating miRNA-based biomarkers into personalized secondary prevention strategies for ischemic stroke [53].

## 5. Conclusions

This study provides novel evidence that circulating microRNAs—specifically miR-126-3p, miR-199a-5p, and miR-223-3p—are significantly associated with antiplatelet therapy efficacy and clinical outcomes in post-stroke patients. Elevated levels of miR-126-3p and miR-199a-5p, particularly in patients on dual antiplatelet therapy and statins, suggest a protective role through modulation of vascular homeostasis and inflammation. In contrast, decreased miR-223-3p levels independently predict recurrent thrombotic events, underscoring its importance in platelet regulation and long-term stroke prevention. Our findings also demonstrate the added value of fractionated plasma analysis using gravity sedimentation to capture dynamic molecular processes beyond conventional clinical risk scores [54].

## Figures and Tables

**Table 1:** Baseline demographic and clinical characteristics of the study cohort.

	Total *n* = 60	ASA *n* = 20	Clopidogrel *n* = 20	DAPT *n* = 20	*p*
Demography					
Age, years	68 ± 11	69 ± 12	68 ± 10	68 ± 11	0.940
F/M, N	24/36	10/10	9/11	5/15	0.233
Clinical features					
Hypertension, N (%)	55 (92)	19 (95)	17 (85)	19 (95)	0.418
Diabetes, N (%)	25 (42)	7 (35)	7 (35)	11 (55)	0.034
Smoking, N (%)	11 (18)	5 (25)	4 (20)	2 (10)	0.459
Hyperlipidemia, N (%)	25 (42)	5 (25)	11 (55)	9 (45)	0.147
Vascular episodes	15 (25)	2 (10)	7 (35)	6 (30)	0.155
Vascular episodes (FU)	7 (12)	1 (5)	3 (15)	3 (15)	0.524
PLTF	254 (213–306)	248 (219–283)	281 (243–339)	221.5 (194.5–326)	0.136
miR-126-3p	1.97 (1.00–2.65)	0.829 (0.379–1.00)	2.00 (1.18–2.57)	2.59 (1.99–3.20)	<0.001 *
miR-223-3p	12.13 (8.26–14.23)	14.05 (5.54–16.77)	9.39 (6.19–12.47)	12.36 (11.56–13.85)	0.014 ^#^
miR-24-3p	0.81 (0.55–0.96)	0.92 (0.80–1.14)	0.44 (0.36–0.54)	0.85 (0.70–0.96)	<0.001 ^†^
miR-199a-5p	0.069 (0.033–0.109)	0.064 (0.035–0.11)	0.031 (0.025–0.046)	0.093 (0.06–0.14)	0.006 ^##^

Number *n*; *p*-value indicates intergroup comparison; F/M female/male; Vascular episodes mean more than one thromboembolic event; PLTF—Platelet Count measured with fluorescent method; FU—18 month follow up study. PLTF and miRNAs are presented as median and 25–75 percentiles (IQR). One-way Anova test: ^#^ C vs. DAPT; * A vs. C, A vs. DAPT, C vs. DAPT; ^†^ A vs. C, A vs. DAPT; ^##^ A vs. DAPT.

## Data Availability

The raw data supporting the conclusions of this article will be made available by the authors on request.
